# Hypoxia‐inducible factor‐1 *α* deletion in myeloid lineage attenuates hypoxia‐induced pulmonary hypertension

**DOI:** 10.14814/phy2.14025

**Published:** 2019-03-29

**Authors:** Hiroshi Kojima, Tomotake Tokunou, Yusuke Takahara, Kenji Sunagawa, Yoshitaka Hirooka, Toshihiro Ichiki, Hiroyuki Tsutsui

**Affiliations:** ^1^ Department of Cardiovascular Medicine Kyushu University Graduate School of Medical Sciences Fukuoka Japan; ^2^ Center for Disruptive Cardiovascular Medicine Department of Advanced Cardiovascular Regulation and Therapeutics Kyushu University Fukuoka Japan; ^3^ Department of Internal Medicine Kyushu University Beppu Hospital Oita Japan; ^4^ International University of Health and Welfare Fukuoka Japan; ^5^ Department of Cardiology Harasanshin Hospital Fukuoka Japan

**Keywords:** Hypoxia, hypoxia‐inducible factor, macrophages, pulmonary hypertension

## Abstract

Hypoxemia is seen in patients with pulmonary hypertension and hypoxic pulmonary vasoconstriction worsens their clinical condition. However, vasoconstriction is not the only aspect through which hypoxia induces the progression to pulmonary hypertension. Hypoxia‐inducible factor‐1*α* (HIF‐1*α*) is a transcription factor responding to hypoxic conditions by regulating hundreds of genes involved in angiogenesis, erythropoiesis, inflammation, and proliferation. We sought to determine the contribution of HIF‐1*α* in myeloid lineage cells to the pulmonary vascular response to chronic exposure to hypoxia. We generated myeloid‐specific HIF‐1*α* knockout (MyeHIF1KO) mice by using Cre‐lox P system, and exposed them to hypoxic conditions for 3 weeks to induce pulmonary hypertension. Macrophages from MyeHIF1KO and control mice were used for western blotting, RT‐qPCR, chemotaxis assay, and ATP assay. MyeHIF1KO mice exposed to hypoxia for 3 weeks exhibited a significant reduction in the right ventricular systolic pressure accompanied by a decrease in the ratio of the right ventricular weight to left ventricular weight, muscularization of the small pulmonary arteries, and infiltration of macrophages into the lung and right ventricle compared with control mice. HIF‐1*α*‐deficient peritoneal macrophages showed less migration toward monocyte chemoattractant protein‐1 and a decrease in intracellular ATP levels. These results indicate that HIF‐1*α* in macrophages contributes to the progression of pulmonary vascular remodeling and pulmonary hypertension induced by chronic exposure to hypoxic conditions. The inhibition of myeloid‐specific HIF‐1*α* may be a novel therapeutic strategy for the treatment of pulmonary hypertension.

## Introduction

The REVEAL registry in 2012 showed that the current 5‐year survival rate for patients with pulmonary arterial hypertension significantly improved (Benza et al. 2012) compared with that reported in the National Institutes of Health Registry in 1991 (D'Alonzo et al. 1991). This improvement is due to the recent clinical application of prostacyclin analogues, endothelin receptor blockers, and phosphodiesterase 5 inhibitors to the treatment of pulmonary hypertension (Wu et al. 2013). These drugs reduce pulmonary arterial pressure through vasodilation of pulmonary artery and attenuation of vascular remodeling. The prognosis of patients with pulmonary hypertension, however, is still poor, with a 5‐year mortality rate exceeding 40% (Benza et al. 2012). In COMPERA registry, 5‐year mortality of pulmonary arterial hypertension is range from 24% (low‐risk group) to 68% (high‐risk group) (Hoeper et al. 2017).

Recent studies have significantly improved our understanding regarding the mechanism underlying the development of pulmonary hypertension. Endothelial damage observed in patients with pulmonary hypertension induces the secretion of vasoconstrictors such as endothelin‐1 and thromboxane A_2_, and reduces the secretion of vasodilators such as nitric oxide and prostaglandin I_2_ (Christman et al. 1992; Giaid et al. 1993). Endothelial dysfunction induces constriction, vascular remodeling, and obliteration of pulmonary arteries, resulting in an increase in the pulmonary arterial pressure, and hypoxia (Christman et al. 1992; Rubens et al. 2001; Humbert et al. 2019). Hypoxemia, which is seen in patients with pulmonary hypertension (Rubin 1997), also leads to vasoconstriction and pulmonary arterial pressure elevation. Increased pulmonary arterial pressure induces the muscularization of distal pulmonary arteries, which causes vicious cycles of elevated pulmonary vascular resistance (Lai et al. 2014). Hypoxia also induces monocyte/macrophage infiltration into pulmonary arterial adventitia (Stenmark et al. 2006). The infiltrated macrophages produce cytokines, chemokines, and growth factors that accelerate pulmonary vascular remodeling and pulmonary hypertension (Stenmark et al. 2006).

Hypoxia‐inducible factor‐1*α* (HIF‐1*α*) is a transcription factor that regulates the cellular responses to hypoxia (Palazon et al. 2014). Under normoxic conditions, the prolyl hydroxylation of HIF‐1*α* by prolyl‐hydroxylase‐domain‐containing proteins (PHDs) leads to the binding of von Hippel–Lindau tumor‐suppressor protein and recruitment of an ubiquitin–ligase complex to HIF‐1*α* (Appelhoff et al. 2004). This complex leads to the degradation of HIF‐1*α* by the 26S proteasome. PHD activity is inhibited under hypoxic conditions, thereby stabilizing the HIF‐1*α* protein. Stabilized HIF‐1*α* activates the expression of the genes involved in glycolysis, angiogenesis, erythropoiesis, and cell survival (Nakayama et al. 2004). Tuder et al. (2001) reported that HIF‐1*α* was expressed in the macrophages of lungs of patients with pulmonary hypertension, especially in plexiform lesions. However, to what extent macrophages and HIF‐1*α* in macrophages affect the progression of pulmonary hypertension has not yet been clarified. Therefore, we sought to determine whether HIF‐1*α* in macrophages is involved in pulmonary arterial remodeling in the process of the progression of pulmonary hypertension. For this purpose, we investigated the effects of the myeloid‐lineage‐specific deletion of HIF‐1*α* on hypoxia‐induced pulmonary hypertension in this study.

## Materials and Methods

### Materials

Dulbecco's Modified Eagle Medium (DMEM) and fetal bovine serum (FBS) were purchased from GIBCO‐BRL, Invitrogen Co. (Carlsbad, CA, USA). Thioglycollate was purchased from Becton Dickinson and Co. (Franklin Lakes, NJ, USA). Cobalt chloride (CoCl_2_) was purchased from Sigma‐Aldrich Co. (C8611‐25G St. Louis, MO, USA). Complete Protease Inhibitor Cocktail solution and RNA isolation kit were purchased from Roche Applied Science (Basel, Switzerland). Bicinchoninic acid protein assay kit was purchased from Thermo Sientific Co. (Waltham, MA, USA). Mini‐PROTEAN TGX Gels (4–20%) were purchased from Bio Rad Co. (Hercules, CA, USA). Polyvinylidene difluoride membrane was purchased from Immobilon‐P, Millipore Co. (Darmstadt, Hessen, Germany). Polyclonal antibodies against HIF‐1*α* were purchased from Novus Biologicals Inc. (Minneapolis, MN, USA). An antibody against Histone H3 was purchased from Cell Signaling Technology, Inc. (Danvers, MA, USA). ECL Prime Western Blotting Detection Reagent was purchased from GE Healthcare (Little Chalfont, Buckinghamshire, England). An antibody against *α* smooth muscle actin (*α*‐SMA) was purchased from Dako (Glostrup, Denmark). An antibody against galectine‐3 (MAC2) was purchased from Cedarlane Co. (Burlington, NC, USA). Simple Stain MAX‐PO (Rat) was purchased from Nichirei Bioscience (Tokyo, Japan). Formaldehyde and 3, 3‐Diaminobensidine (DAB) were purchased from Wako (Osaka, Japan). Revertra Ace was purchased from Toyobo Co. (Osaka, Japan). Thunderbird SYBR Green PCR Master mix was purchased from Life Technologies (Tokyo, Japan).

### Isolation of peritoneal macrophages

To collect peritoneal macrophages (PMs), mice were intraperitoneally injected with 2.0 mL of 3% thioglycollate. Four days later, the peritoneal cavity was lavaged with 10 mL phosphate buffered saline (PBS) to retrieve the infiltrated cells. After centrifugation (89 *g*, 5 min, 4°C), the pelleted cells were resuspended in DMEM containing 10% FBS, seeded in a cell culture dish and incubated at 37°C with 5% CO_2_ overnight. Nonadherent cells were removed by washing with PBS, and adherent cells were used as PMs (Ikeda et al. 2013). PMs in DMEM with 1% FBS were exposed to normoxic (21% O_2_) or hypoxic (1%O_2_) conditions and used for experiments.

### Western blot analysis

To prepare nuclear protein, PMs were suspended in a buffer containing 10 mmol/L HEPES‐KOH (pH 7.9), 1.5 mmol/L MgCl_2_, 10 mmol/L KCl, 0.5 mmol/L DTT, 0.2 mmol/L phenylmethylsulfonyl fluoride, 0.2 mmol/L CoCl_2_, 1 × Complete Protease Inhibitor Cocktail solution, and 0.6% NP‐40. Nuclei were pelleted by centrifugation (1000 *g*), and the nuclear proteins were exacted with a buffer containing 20 mmol/L HEPES‐KOH, 1.5 mmol/L MgCl_2_, 420 mmol/L NaCl, 0.5 mmol/L DTT, 0.2 mmol/L CoCl_2_, 25% glycerol, and 1 × Complete Protease Inhibitor Cocktail solution. Nuclear extracts were cleared by centrifugation and used for a Western blot analysis. Protein concentrations were determined with the bicinchoninic acid protein assay kit. Cell lysates were heated in a sample buffer (62.5 mmol/L Tris‐HCl [pH 6.8], 10% glycerol, 2% SDS, 0.05% bromophenolblue, and 715 mmol/L 2‐mercaptoethanol) at 95°C for 3 min, electrophoresed on 4–20% Mini‐PROTEAN TGX Gels, and transferred to polyvinylidene difluoride membrane. The blots were blocked with TBS‐T (20 mmol/L Tris‐HCl [pH 7.6], 137 mmol/L NaCl, 0.1% Tween 20) containing 5% skim milk at room temperature for 30 min. Blots were detected using a chemiluminescence system with ECL Prime Western Blotting Detection Reagent. The protein expression was quantified by ImageQuant LAS 4010 (GE Healthcare, Little Chalfont, Buckinghamshire, England) (Ikeda et al. 2013).

### Quantitative real‐time reverse transcription polymerase chain reaction (RT‐PCR)

Total mRNA was isolated from PMs exposed to normoxia or hypoxia for 8 h using an RNA isolation kit (Roche) in accordance with the manufacturer's instructions and reversed transcribed using ReverTra Ace. cDNA (700 ng) was subjected to a real‐time polymerase chain reaction (PCR) using Thunderbird SYBR qPCR Mix with a 7500 real time PCR system (Applied Biosystems, Foster City, CA, USA) for quantitative real‐time reverse transcription PCR in the Research Support Center, Research Center for Human Disease Modeling, Kyushu University Graduate School of Medical Sciences.

The specificity of the amplified PCR product was verified by a melting curve analysis. The relative mRNA levels were analyzed via the quantitative comparative Ct method and normalized with 18s gene expression as an internal control (Ikeda et al. 2015). The nucleotide sequences of primers are used:

*Arginase 1 (Arg1)*, Forward (F); 5′‐AGCTCTGGGAATCTGCATGG‐3′,

Reverse (R); 5′‐ ATGTACACGATGTCTTTGGCAGATA‐3′,

*Glucose transporter 1 (Glut1)*, F; 5′‐ CTTCATTGTGGGCATGTGCTTC‐3′,

R; 5′‐ AGGTTCGGCCTTTGGTCTCAG‐3′,

*Hif1a*, F; 5′‐ TGAGCTTGCTCATCAGTTGC‐3′, R; 5′‐ CCATCTGTGCCTTCATCTCA‐3′,

*Interleukin 1β (Il1b)*, F; 5′‐ TCCAGGATGAGGACATGAGCAC‐3′, R; 5′‐ GAACGTCACACACCAGCAGGTTA‐3′,

*Nitric oxide synthase 2 (iNos)*, F; 5′‐CAAGCTGAACTTGAGCGAGGA‐3′,

R; 5′‐TTTACTCAGTGCCAGAAGCTGGA‐3′,

*Platelet derived growth factor B (Pdgfb)*, F; 5′‐ CAAAGGCAAGCACCGAAAGTTTA‐3′, R; 5′‐ CCGAATCAGGCATCGAGACA‐3′,

*Vascular endothelial growth factor a (Vegfa)*, F; 5′‐GCACATAGGAGAGATGAGCTTCC‐3′, R; 5′‐ CTCCGCTCTGAACAAGGCT‐3′.

### Animal experiments

**Figure 1:**
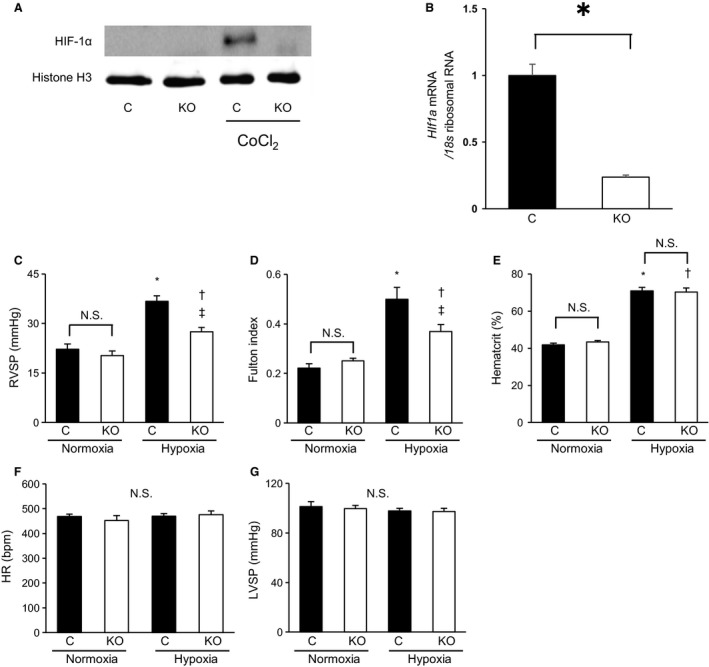
The hypoxia‐induced RVSP elevation and right ventricular hypertrophy were suppressed by MyeHIF1KO. (A, B) Confirmation of myeloid‐specific HIF‐1*α* knockout by Western blot analysis and RT‐PCR. A, A representative image of HIF‐1*α* expression relative to Histon H3 expression by Western blot analysis is shown. Peritoneal macrophages (PMs) from control mice or MyeHIF1KO mice were used. Peritoneal macrophages were incubated with or without cobalt chloride (CoCl_2_, 100 *μ*mol/L), a PHD inhibitor for 4 h, nuclear proteins were isolated and exmained by Western blot analysis. B, mRNA expression of HIF‐1*α* in PMs from control mice or MyeHIF1KO mice was examined by quantitaive RT‐PCR. Expression of HIF‐1*α* was normalized by that of 18s ribosomal RNA. *n* = 3. * *P* < 0.01. C–G, (C) right ventricular systolic pressure (RVSP), (D) Fulton index: right ventricular weight (RVW) standardized by the left ventricular and interventricular septum weight, (E) Hematocrit, (F) heart rate (HR), and (G) left ventricular systolic pressure (LVSP) of control mice or MyeHIF1KO mice in normoxia or hypoxia (10% O_2_ for 3 weeks) models. (C) *n* = 5 (C+normoxia), 6 (KO+normoxia), 8 (C+hypoxia), 8 (KO+hypoxia), (D) *n* = 7 (C+normoxia), 6 (KO+normoxia), 6 (C+hypoxia), 8 (KO+hypoxia), (E) *n* = 6 (C+normoxia), 6 (KO+normoxia), 6 (C+hypoxia), 8 (KO+hypoxia), (F) 5 (C+normoxia), 6 (KO+normoxia), 8 (C+hypoxia), 8 (KO+hypoxia), (G) *n* = 5 (C+normoxia), 6 (KO+normoxia), 5 (C+hypoxia), 4 (KO+hypoxia). * *P* < 0.05 versus control mice in normoxia. †*P* < 0.05 versus KO mice in normoxia. ‡ *P* < 0.05 versus control mice in hypoxia. C, control; KO, MyeHIF1KO; N.S. not significant

All animal experiments were approved by the Animal Care and Use Committee, Kyushu University and conducted in accordance with the institutional guidelines. Myeloid‐specific HIF‐1*α*‐deficient (MyeHIF1KO) mice on a C57BL/6 background were generated by using Cre lox P recombination system. The expression of Cre recombinase was driven by the promotor of lysozyme M (LysM) (Clausen et al. 1999). LysM Cre mice with C57BL6 background were purchased from Riken Bio Resource Center (Ibaraki, Japan). First, we crossed homozygous HIF1*α*‐floxed (Hif1*α* ^flox/flox^) mice (Jackson Laboratory, Bar Harbor, Maine, USA) and LysM Cre mice to create heterozygous HIF1*α* ^flox/+^ mice with LysM Cre. Then, we created MyeHIF1KO (HIF1*α* ^flox/flox^ with LysM Cre) mice by crossing HIF1*α* ^flox/flox^ mice and HIF1*α* ^flox/+^ mice with LysM Cre. HIF‐1*α* ^flox/flox^ mice were used as control mice. Male mice were used for our experiments. We confirmed myeloid specific HIF1*α* knockout by Western blot analysis and RT‐PCR (Fig. 1A, B). To create a hypoxic environment, adult male mice (20–26 g, 7 weeks) were kept in a ventilated chamber (TEIJIN, Tokyo, Japan) that provided a constant O_2_ level of 10% for 3 weeks (Chen et al. 2015; Ten Freyhaus et al. 2015). PowerLab (AD Instruments, Bella Vista, New South Wales, Australia) was used for the hemodynamic analysis.

### Hemodynamic analysis

Each mouse was anesthetized by isoflurane inhalation via a face mask. An isoflurane gas machine was connected to the mice via a tube. Isoflurane gas was mixed with air or 10% O_2_. To evaluate the pulmonary arterial pressure, the right ventricle was punctured at a subxiphoid site using a percutaneously inserted 25 gauge needle connected to a pressure transducer (Eddahibi et al. 2000), and the right ventricular systolic pressure (RVSP) and heart rate (HR) were measured by PowerLab (AD Instruments) (Mohammadi et al. 2012). After the needle penetrated the intraventricular septum, the left ventricular systolic pressure (LVSP) was measured. After the hemodynamic measurements, mice were injected ketamine (80 mg/kg) and xylazine (10 mg/kg) with their peritoneal cavity (Eddahibi et al. 2000), their chests were opened, and the blood was cleared from their lungs and hearts with PBS via the right ventricle using a 22‐gauge needle. The lung tissues were collected for histological and molecular analyses. The heart was removed. The right ventricle was dissected from the left ventricle to analyze the right ventricular weight (RVW) (Vitali et al. 2014).

### Histological analysis

The left lung was inflated with 0.5% agarose and 1% formalin via the trachea (Homma et al. 2008). The left lung was then removed, fixed in 10% formaldehyde overnight, and embedded into paraffin via a standard procedure. The left lung was dissected transversely into two pieces that were arranged in sequence and embedded in the same paraffin cassette and cut at 5‐*μ*m thickness before being placed onto glass slides. Lung specimens were de‐paraffinized in a series of xylene baths. The slides were then rehydrated in graded ethanol and distilled water. To retrieve antigenicity, the tissue sections were incubated in citrate buffer at 121°C for 20 min. After cooling at room temperature for 30 min, the sections were washed with PBS and immersed in PBS containing 3% hydrogen peroxide (H_2_O_2_) for 20 min. For *α*‐SMA or MAC2 Primary antibody was then applied, and the sections were incubated at 4°C overnight, after which secondary antibody was applied for 1 h at room temperature. DAB was used as a chromogen (Ten Freyhaus et al. 2015).

### Classification of muscularization of small pulmonary artery

We created three categories to describe the muscularization of small pulmonary arteries (<70 *μ*m), in accordance with the methods of previous studies (Yamazato et al. 2009). Based on the circumferential staining of *α*‐SMA, a vessel was deemed nonmuscularized (<25% staining), partially muscularized (25%‐75% staining), or fully muscularized (>75% staining) (Xia et al. 2014; Ten Freyhaus et al. 2015).

### Assessment of macrophage infiltration into lung and right ventricle

To investigate the macrophage infiltration around pulmonary arteries for early‐stage pulmonary hypertension, we counted the number of MAC2‐positive macrophages around 40 small pulmonary arteries (<70 *μ*m) for each section of mouse lung exposed to normoxic or hypoxic conditions for 3 days (Hashimoto‐Kataoka et al. 2015). Macrophage infiltration into the right ventricle was also assessed by counting the number of MAC2‐positive macrophages per field in the right ventricles exposed to normoxic or hypoxic conditions for 3 weeks.

### Chemotaxis assay

A chemotaxis assay was performed with 96‐well ChemoTx System (Neuro Probe Inc. Gaithersburg, MD, USA) using isolated PMs in accordance with the manufacturer's instruction. PMs (2 × 10^6^ cells/mL) in DMEM containing 0.1% BSA were loaded into the upper wells. The lower wells separated by polycarbonate membrane with 8‐*μ*m pores were filled with the same medium containing monocyte chemotactic protein‐1 (MCP‐1) (0, 10, 25 ng/mL). After incubation for 16 h at 37°C under normoxic or hypoxic conditions, the number of migrated cells per field on the lower surface of the membrane was counted after staining with Diff‐Quik (Sysmex, Kobe, Japan). Experiments were performed in duplicate and repeated at least three times (Ikeda et al. 2013).

### Measurement of intracellular ATP

To estimate the macrophage metabolism, intracellular ATP was measured by a luciferin‐luciferase bioluminescence assay using a cellular ATP assay reagent (Toyo‐Ink, Tokyo, Japan). The macrophages were seeded at 3 x 10^4^ cells/100 *μ*L/well in a 96‐well tissue culture plate and incubated in a CO_2_ incubator at 37°C for 4 h under normoxic conditions and then for additional 1 h under normoxic or hypoxic conditions. The plate was incubated at room temperature for 30 min, and 100 *μ*L ATP reagent was then added to each well. The plate was stirred for 1 min and then incubated in a luminometer, Insight (Perkin Elmer, Waltham, MA, USA) for 10 min at 23°C. The relative light intensity was then measured (Nishi et al. 2010).

### Statistical analysis

The experimental data were analyzed by one‐way ANOVA and Tukey's honestly significant difference test or Student's *t*‐test when data were confirmed as normally distributed with similar variance between comparator groups. The results are shown as the mean±standard error. *P < *0.05 was considered statistically significant.

## Results

### Attenuation of hypoxia‐induced pulmonary hypertension in MyeHIF1KO mice

To clarify the role of macrophage HIF‐1*α* in the development of pulmonary hypertension, control mice and MyeHIF1KO mice were exposed to hypoxic conditions (10% O_2_) for 3 weeks. Table 1 shows the baseline and final body weights after 3 weeks of exposure to hypoxic conditions. The body weight of mice under normoxic conditions was increased, whereas that of mice under hypoxic conditions was decreased. However, there were no significant differences in the body weights between the control mice and MyeHIF1KO mice under normoxic or hypoxic conditions.

**Table 1:** Body weight (BW) before and after three weeks of exposure to normoxia or hypoxia

	Normoxia		Hypoxia	
	Control	MyeHIF1KO	Control	MyeHIF1KO
	(*n* = 7)	(*n* = 6)	(*n* = 8)	(*n* = 8)
Baseline BW (g)	24.5 ± 0.4	24.2 ± 0.5	23.6 ± 0.6	24.0 ± 0.5
Final BW (g)	27.6 ± 0.3*	27.7 ± 0.3*	21.3 ± 0.6*	21.1 ± 0.7*

BW, body weight.

The data are expressed as the mean ± SEM (*n* = 6–8). *P < 0.05 versus baseline.

Control mice showed a significant RVSP elevation (36.7 ± 1.6 mmHg vs. 22.2 ± 1.5 mmHg in normoxia; *P* < 0.05) after 3 weeks of exposure to hypoxic conditions. There was no significant difference in RVSP between control mice and MyeHIF1KO mice under normoxia. However, the hypoxia‐induced RVSP elevation was significantly suppressed in MyeHIF1KO mice (27.5 ± 1.3 mmHg vs. 36.7 ± 1.6 mmHg in control mice under hypoxia; *P* < 0.05, Fig. 1C). The ratio of RVW to left ventricular weight (LVW), including the interventricular septum weight (Fulton index), was significantly increased in control mice under hypoxia (0.50 ± 0.05 vs. 0.22 ± 0.02 under normoxia; *P* < 0.05). There was no significant difference in the Fulton index between control mice and MyeHIF1KO mice under normoxia. However, the hypoxia‐induced increase in Fulton index was significantly suppressed in MyeHIF1KO mice (0.38 ± 0.02 in MyeHIF1KO mice under hypoxia. vs. 0.50 ± 0.05 in control mice under hypoxia; *P* < 0.05, Fig. 1D). These data suggest that deletion of HIF‐1*α* in myeloid lineage significantly suppressed hypoxia‐induced right ventricular hypertrophy (Fulton index). The hematocrit level was increased by hypoxia in both control mice and MyeHIF1KO mice. However, the hypoxia‐induced hematocrit level was not different between control mice and MyeHIF1KO mice (Fig. 1E). The HR and LVSP between control mice and MyeHIF1KO mice were also equivalent (Fig. 1F, G).

### Attenuation of hypoxia‐induced pulmonary vascular muscularization in MyeHIF1KO mice

**Figure 2:**
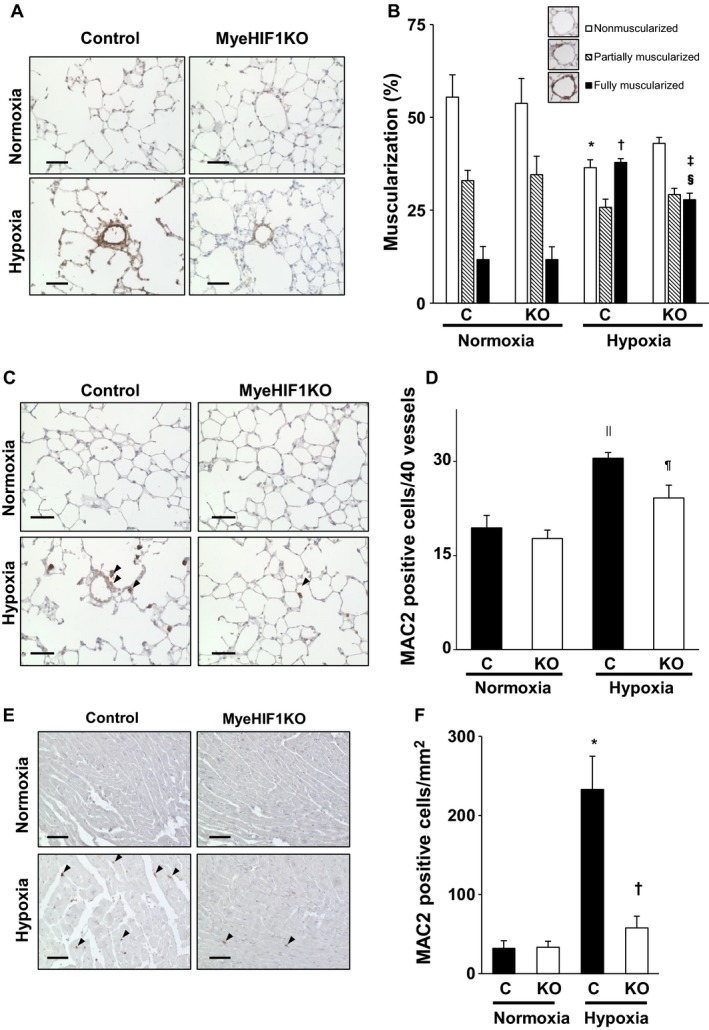
Muscularization in the distal pulmonary arteries after 3 weeks under hypoxic conditions. Macrophage infiltration into the lung after 3 days. (A) Representative *α*‐SMA‐stained mouse lung tissue (brown) of control mice or MyeHIF1KO mice in normoxia or hypoxia (10%, 3 weeks). Scale bars are 50 *μ*m. (B) The percentage of hypoxia‐induced muscularization of distal pulmonary arteries (<70 *μ*m diameter) by characterization as nonmuscularized, partially muscularized, and fully muscularized. Forty vessels were counted per sample. (C) Representative microphotographs of lung immunohistochemistry for MAC2‐positive macrophages (brown) after 3 days exposure to hypoxia (10%) or normoxia are shown. Scale bars are 50 *μ*m. The arrowheads indicate representative MAC2‐positive cells. (D) The number of MAC2‐positive macrophages around the pulmonary arteries of 40 vessels from each sample. (B), *n* = 6 (C+normoxia), 6 (KO+normoxia), 7 (C+hypoxia), 6 (KO+hypoxia). (D), *n* = 8 (C+normoxia), 7 (KO+normoxia), 8 (C+hypoxia), 7 (KO+hypoxia). * *P* < 0.05 versus nonmuscularized for control mice in normoxia. † *P* < 0.05 versus fully muscularized for control mice in normoxia. ‡ *P* < 0.05 versus fully muscularized for KO mice in normoxia. § *P* < 0.05 versus fully muscularized for control mice in hypoxia. MyeHIF1KO mice. || *P* < 0.05 versus control mice in normoxia. ¶ *P* < 0.05 versus control mice in hypoxia. C, control mice; KO, MyeHIF1KO mice. (E) Representative microphotographs of immunohistochemical analysis for MAC2‐positive macrophages in the right ventricle (brown) after 3 weeks’ exposure to hypoxia (10%) or normoxia are shown. Scale bars are 50 *μ*m. The arrowheads indicate MAC2‐positive cells. (F) The number of MAC2‐positive cells per square millimeter from each sample are counted and indicated in bar graphs. *n* = 6 (C+normoxia), 7 (KO+normoxia), 6 (C+hypoxia), 7 (KO+hypoxia). * *P* < 0.05 versus control mice in normoxia. † *P* < 0.05 versus control mice in hypoxia. C, control mice; KO, MyeHIF1KO mice.

We assessed hypoxia‐induced hypertrophy of the pulmonary vessel wall by staining with anti‐*α*‐SMA antibody (Fig. 2A). The percentage of nonmuscularized artery was more than 50%, and that of partially muscularized artery was more than 30%, with no significant difference between control and MyeHIF1KO mice in normoxic conditions (Fig. 2B). The proportion of fully muscularized arteries in the control mice after exposure to hypoxic conditions for 3 weeks was significantly increased compared with the baseline values (Fig. 2A lower panel), whereas that in MyeHIF1KO mice was significantly attenuated (27.8%±1.8%, vs. 37.8%±1.0% in control mice in hypoxia; *P* < 0.05, Fig. 2B).

### Attenuation of hypoxia‐induced macrophage infiltration into pulmonary artery in MyeHIF1KO mice

Figures 2C and D show that exposure to hypoxic conditions for 3 days increased the infiltration of MAC2‐positive macrophages around pulmonary arteries in control mice (30.5 ± 0.9/40 vessels vs. 19.4 ± 2.0/40 vessels of control mice in normoxia; *P* < 0.05). The hypoxia‐induced infiltration of MAC2‐positive macrophage was attenuated in MyeHIF1KO mice compared with control mice (24.1 ± 2.0/40 vessels vs. 30.5 ± 0.9/40 vessels in control mice in hypoxia; *P* < 0.05). Under normoxic conditions, very few MAC2‐positive macrophages were observed in the right ventricle of control mice (31.8 ± 9.9/mm^2^) and MyeHIF1KO mice (33.1 ± 7.8/mm^2^). Under hypoxic condition, the number of MAC2‐positive macrophages was increased in control mice, and this increase was significantly attenuated in MyeHIF1KO mice (57.6 ± 14.7/mm^2^ vs. 233.1 ± 41.8/mm^2^ in control mice, *P* < 0.05) (Fig. 2E, F).

### Gene expression of MyeHIF1KO macrophages

**Figure 3:**
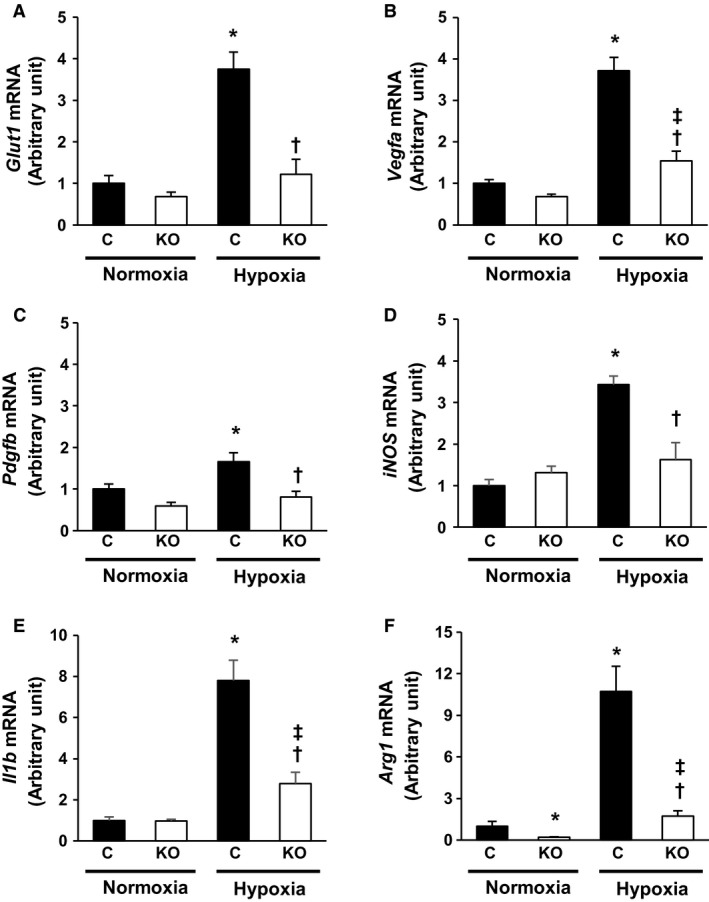
The gene expression of PMs under hypoxic condition. A‐D, (A) *Glut1* mRNA, (B) *Vegfa* mRNA, (C) *Pdgfb* mRNA, (D) *iNos* mRNA, (E) *Il1b* mRNA, (F) *Arg1* mRNA as the relative mRNA level to 18s ribosomal RNA in PMs derived from control or MyeHIF1KO mice exposed to normoxic or hypoxic conditions (1% O_2_) for 8 h. *n* = 9 (C+normoxia), 7 (KO+normoxia), 8 (C+hypoxia), 7 (KO+hypoxia). * *P* < 0.05 versus PMs derived from control mice in normoxia. † *P* < 0.05 versus PMs derived from control mice in hypoxia. ‡ *P* < 0.05 versus PMs derived from KO mice in normoxia. C, control mice; KO, MyeHIF1KO mice

We next investigated the expression of HIF‐1*α* target genes, such as *Glut1*,* Vegfa*,* Pdgfb*,* Il1b*,* iNos,* and *Arg1* in macrophages. The expression of *Glut1*,* Vegfa*, and *Pdgfb* was significantly upregulated by hypoxia (8 h) in PMs from control mice; however, the upregulation was significantly attenuated in PMs from MyeHIF1KO mice (Fig. 3A–C). mRNA expression of M1 and M2 markers was examined. The mRNA expression of *Il1b* and *iNos* as M1 markers was significantly increased in control mice and was not increased in MyeHIF1KO mice under hypoxic conditions (Fig. 3D, E). *Arg1* as an M2 marker was also significantly increased in control mice and suppressed in MyeHIF1KO mice under hypoxic conditions (Fig. 3F). However, hypoxic condition did not increase the mRNA expression of other M1 markers (*tumor necrosis factor alpha*,* Mcp‐1*) or M2 markers (*found in inflammatory zone‐1*,* mannose receptor C type 1*) in both control and MyeHIF1KO mice (data not shown).

### Attenuation of macrophage migration in HIF‐1α‐deficient mice

**Figure 4:**
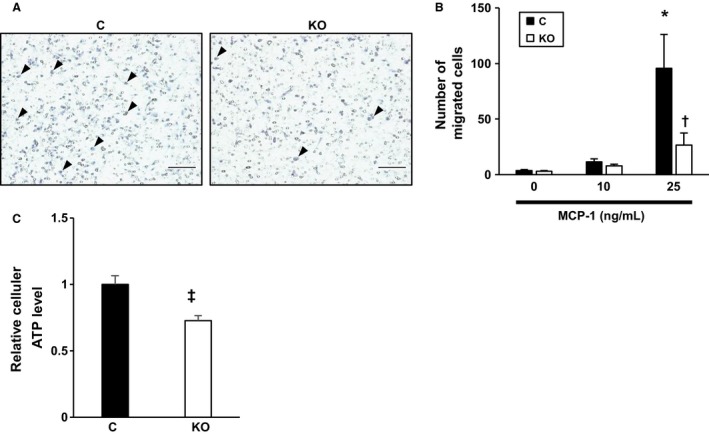
The attenuation of macrophage migration in HIF‐1*α*‐deficient mice. (A) Representative images of a macrophage chemotaxis assay induced by MCP‐1 (25 ng/mL) under hypoxic conditions (1% O_2_) for 16 h are shown. Scale bar is 100 *μ*m. The arrowheads indicate migrated macrophages. (B) A quantitative analysis of the macrophage chemotaxis assay (*n* = 5) by MCP‐1 (0, 10, 25 ng/mL) under hypoxic conditions. Number of migrated cells per low‐power field was counted. (C) The intracellular ATP concentrations of macrophages under hypoxic conditions (1% O_2_) measured by a luciferase‐based chemiluminescent assay (*n* = 6) are shown. * *P* < 0.05 versus PMs derived from control mice with 0 ng/mL MCP‐1. † *P* < 0.05 versus PMs derived from control mice with 25 ng/mL MCP‐1. ‡ *P* < 0.05 versus PMs derived from control mice in normoxia. § *P* < 0.05 versus PMs derived from control mice in hypoxia. C, control; KO, MyeHIF1KO.

We then examined macrophage migration induced by MCP‐1. MCP‐1 dose‐dependently increased the number of migrated PMs obtained from control and MyeHIF1KO mice. The migration of PMs from MyeHIF1KO mice was significantly suppressed under hypoxic conditions (Fig. 4A, B). Cellular ATP levels were significantly lower in PMs from MyeHIF1KO mice than in those from control mice under hypoxic conditions (Fig. 4C).

## Discussion

In this study, we showed for the first time that the myeloid‐lineage specific deletion of HIF‐1*α* attenuated hypoxia‐induced increase in RVSP and RV hypertrophy in association with a reduction in the macrophage infiltration into the lung and right ventricle. Our findings suggest that HIF‐1*α* in myeloid lineage cells is involved in the development of hypoxia‐induced pulmonary hypertension. In addition, the hypoxia‐induced vascular remodeling process, that is, the muscularization of distal pulmonary arteries that are mainly responsible for pulmonary artery resistance (Pugliese et al. 2015), associated with the numbers of hypoxia‐induced macrophages infiltration.

The mechanism of reduced macrophage motility has not been clarified in this study. However, a reduction in ATP production in PMs from MyeHIF1KO mice may be one of the possible mechanisms underlying the reduction in macrophage migration in MyeHIF1KO mice, because the migration of macrophages requires energy. The oxygen tension is reduced and glycolysis is activated by HIF‐1*α* as macrophages migrate into the organs. Insufficient induction of glycolytic enzymes and glucose transporter (Glut1) in HIF‐1*α*‐deficient cell may cause insufficient glucose uptake and glycolysis and thereby result in the reduction in the intracellular ATP levels (Freemerman et al. 2014). Although we have not excluded other possible mechanisms for reduced migration of HIF‐1*α*‐deficient macrophages, the expression of the MCP‐1 receptor C‐C chemokine receptor type 2 did not differ between control mice and MyeHIF1KO mice (data not shown), suggesting that MCP‐1 signaling pathway may be intact. Further study to determine the mechanism of reduced migration of HIF‐1*α*‐deficient macrophages is needed.

In this study, we found that the gene expression of *Vegfa* and *Pdgfb* is upregulated after hypoxic exposure in PMs from control mice but not in those from MyeHIF1KO mice. VEGF is known to increase angiogenesis and macrophage infiltration into pulmonary artery adventitia (Yamaji‐Kegan et al. 2006). Activated *β*‐PDGF receptor by PDGF contributes to proliferation of pulmonary arterial smooth muscle cells (Ten Freyhaus et al. 2015). Thus, the decrease in hypoxia‐induced VEGF and PDGF expression in PMs from MyeHIF1KO mice may play an important role in the attenuation of hypoxia‐induced pulmonary hypertension. SU5416, a VEGF receptor 2 inhibitor, is used to induce pulmonary hypertension in rat, which may not agree with our assumption. However, SU5416 induces apoptosis of endothelial cells, and then apoptosis‐resistant endothelial cells proliferate and induce pulmonary vascular remodeling (Taraseviciene‐Stewart et al. 2001). Because SU5416 is administered by a single injection (Jiang et al. 2016), it is suggested that the effects of relatively long‐term suppression of endogenous VEGF may be different from those of short‐term suppression of VEGF signaling. The role of VEGF in the development of pulmonary hypertension should be addressed in the future study.

A previous study showed that the smooth muscle cell‐specific deletion of HIF‐1*α* in mice also attenuated hypoxia‐induced pulmonary hypertension and vascular remodeling (Ball et al. 2014). However, RV hypertrophy was not affected in these mice after exposure to chronic hypoxia despite a reduction in pulmonary blood pressure, which is apparently inconsistent with our findings that showed RV hypertrophy was also attenuated in MyeHIF1KO mice exposed to chronic hypoxia. The mechanisms for the differential effects of HIF‐1*α* deletion between smooth muscle cells and macrophages on pulmonary hypertension are not clear. This study indicated that less macrophage infiltration into RV in MyeHIF1KO mice after exposure to hypoxic conditions, suggesting that infiltrated macrophages may play some role in the hypoxia‐induced RV hypertrophy. It is also plausible that HIF‐1*α* target genes are different between smooth muscle cells and macrophages and differentially affects RV hypertrophy.

Macrophages release proinflammatory cytokines and growth factors. Based on the expression profiles of cyotokines and growth factors, macrophages are roughly categorized as M1, generally proinflammatory and M2, generally anti‐inflammatory. Several previous reports have shown an increase in the levels of M1 and M2 markers in pulmonary hypertension. Macrophages from pulmonary hypertension upregulated so‐called M1 markers, such as TNF‐*α* and IL‐6, and M2 markers, such as Fizz1 and Arg1 (Albina et al. 1995; Hashimoto‐Kataoka et al. 2015). In our research, some of the M1 marker genes (*Il1b*,* iNos*) and M2 marker gene (*Arg1*) were upregulated under hypoxic conditions, whereas other genes (*Tnfa*,* Il6*,* Mcp1*,* Fizz1*,* Mrc1*) were not increased (data not shown). These results suggest that typical M1/M2 polarization of macrophages might not be involved in our model of pulmonary hypertension.

Recent studies suggest myeloid cells other than macrophages such as neutrophils and mast cells play a role in the development of pulmonary hypertension. Although relatively little attention has been paid to neutrophil, several studies showed the role of neutrophil elastase in the pathogenesis of pulmonary hypertension. Inhibition of neutrophil elastase induces regression of monocrotaline‐induced pulmonary hypertension (Cowan et al. 2000). Mast cells also are derived from myeloid lineage. Mast cells are reported to be observed in perivascular area of pulmonary artery in patients with idiopathic pulmonary arterial hypertension and in a monocrotaline‐induced pulmonary hypertension model (Dahal et al. 2011). Treatment with cromolyn sodium salt that stabilizes and prevents mast cells degranulation attenuated monocrotaline‐induced pulmonary hypertension. Because we focused on the role of macrophages HIF‐1*α* in the development of pulmonary hypertension, we have not examined the role of HIF‐1*α* in other myeloid lineage cells, which is a limitation of this study, and further studies are needed to clarify the role of neutrophil and mast cells HIF‐1*α* in the development of pulmonary hypertension.

We demonstrated that expression of several genes was attenuated by HIF‐1*α* deletion in macrophages (Fig. 3), which may play a critical role in the development of pulmonary hypertension. However, we were unable to determine the most important gene regulated by HIF‐1*α* that is involved in the development of pulmonary hypertension, which we acknowledge as another limitation of this study. Nevertheless, our findings clearly demonstrated that the myeloid cells‐specific deletion of HIF‐1*α* suppressed hypoxia‐induced pulmonary hypertension and myeloid cells played important roles in the progression of pulmonary arterial remodeling. The intervention in HIF‐1*α* pathway may be a novel strategy for the treatment of pulmonary hypertension.

## Conflict of Interest

Center for Disruptive Cardiovascular Medicine Department of Advanced Cardiovascular Regulation and Therapeutics, Kyushu University is supported by Actelion Pharmaceuticals Japan Ltd (T.T. and Y.H.).
